# Effects of Motor Imagery Tasks on Brain Functional Networks Based on EEG Mu/Beta Rhythm

**DOI:** 10.3390/brainsci12020194

**Published:** 2022-01-30

**Authors:** Hongli Yu, Sidi Ba, Yuxue Guo, Lei Guo, Guizhi Xu

**Affiliations:** 1 State Key Laboratory of Reliability and Intelligence of Electrical Equipment, Hebei University of Technology, Tianjin 300130, China; guoshengrui@163.com (L.G.); gzxu@hebut.edu.cn (G.X.); 2 Tianjin Key Laboratory of Bioelectromagnetic Technology and Intelligent Health, Hebei University of Technology, Tianjin 300130, China; bsd21541@126.com (S.B.); guoyuxue178@163.com (Y.G.)

**Keywords:** motor imagery, brain functional network, Granger causality, EEG signal

## Abstract

Motor imagery (MI) refers to the mental rehearsal of movement in the absence of overt motor action, which can activate or inhibit cortical excitability. EEG mu/beta oscillations recorded over the human motor cortex have been shown to be consistently suppressed during both the imagination and performance of movements, although the specific effect on brain function remains to be confirmed. In this study, Granger causality (GC) was used to construct the brain functional network of subjects during motor imagery and resting state based on EEG in order to explore the effects of motor imagery on brain function. Parameters of the brain functional network were compared and analyzed, including degree, clustering coefficient, characteristic path length and global efficiency of EEG mu/beta rhythm in different states. The results showed that the clustering coefficient and efficiency of EEG mu/beta rhythm decreased significantly during motor imagery (*p* < 0.05), while degree distribution and characteristic path length increased significantly (*p* < 0.05), mainly concentrated in the frontal lobe and sensorimotor area. For the resting state after motor imagery, the changes of brain functional characteristics were roughly similar to those of the task state. Therefore, it is concluded that motor imagery plays an important role in activation of cortical excitability.

## 1. Introduction

Motor imagery (MI) is carried out as a mental representation of movement in the absence of corresponding muscle activities [1,2]. When you perform a task of consciousness, such as imagination, the cortex is activated, and the local component of the EEG in this area is attenuated [3]. Recently, cognitive physiology research has shown that the regions used to perform motor tasks might be essentially the same as those activated by motor imagery [4,5]. In addition, similar neural features are observed at the cortical level during both processes. That is, the prefrontal cortex, which is responsible for maintaining and guiding the imaginative motor task, shows selective activation during the task [6]. However, the influence of motor imagery on brain activity remains to be further explored. 

Electroencephalography (EEG) has the advantage of high temporal resolution in brain function analysis, and many studies have used it to analyze the neurophysiological mechanism of EEG rhythm changes related to motor imagery [7]. For EEG rhythm, the cortical regions wherein alpha rhythm is distributed can be divided into two categories: one is alpha oscillation in the sensorimotor cortex region, also known as mu (8–13.0 Hz) rhythm; and the other is alpha rhythm in the visual cortex or occipital lobe. Mu rhythm is recorded in the sensorimotor region, which is not affected by vision, but it changes in response to the preparation of action or motor imagery [8,9]. A portion of the frequencies in beta rhythm (13–30.0 Hz) are harmonics of mu rhythm, which are also related to motor imagery and sensory discrimination [10]. Many groups have studied changes in cortical excitability in different brain regions under motor-imagery-related rhythm and found that the associated representation of motor imagery in mu/beta rhythm might activate or inhibit cortical excitability. Among them, Giromini [11] indicated that significant mu suppression occurred when subjects perceived movement, regardless of the experimental condition. McFarlan [12] mentioned the neurophysiological mechanism of motor imagery ability and found that both motor and imagination movements are related to the desynchronization of mu/beta rhythms, with strong differences in the central region of the right brain. The reduction in brain activity in mu rhythm during the execution of imagination tasks is a weakened version of actual motor execution. Giacomo [13] found significant changes in alpha and beta power in the frontal lobe region when subjects performed hand motion imagination tasks. Cattai [14] also selected the hand imagination task and found that network features based on spectral coherence increased in the sensorimotor region during motor imagery. Although these studies suggest that activation of different brain regions is associated with changes in motor-imagery-related rhythm characteristics, the exact relationship between the two remains unclear.

In recent years, brain research has focused on the characteristics of complex networks, which include anatomical connections, functional connections and effective connections. Effective connectivity provides information about the causal interaction between one neural element or brain region to another neural element or brain region [15]. In applications of brain–machine interfaces, focus should be placed on motor imagery, while the effective connection characteristics of neurophysiology are still poorly understood. Power changes are mostly used to measure the changes of brain regions and rhythm characteristics during motor imagery, and relatively few studies have been conducted on the construction of directed connection networks. Therefore, it is of great significance to discuss the causal relationship between EEG signals and to build a directed connection brain network. In 1956, Wiener proposed a definition of causality between two time-domain signals, and in 1969. Based on this definition, Granger put forward a causality formula of economic stochastic process based, called Granger causality, which was widely applied in the study of brain function connectivity after its development [16]. GC could not only describe the intensity of interaction between different brain regions but also showed the causal relationship between regions, which has the most obvious advantages compared with traditional analysis methods, such as correlation, coherence and mutual information algorithms. Furthermore, the GC algorithm quantifies bi-directional interactions in terms of causality [17]. Therefore, GC analysis of EEG signals, combined with dynamic brain functional networks, could represent the characteristics of EEG signals better in time resolution.

In this study, brain functional networks of motor imagery based on EEG mu/beta rhythm were constructed and analyzed. Degree, clustering coefficient, characteristic path length and global efficiency of mu/beta rhythm of brain functional networks in different states were compared and analyzed. The relationship between the activation of different brain regions and the rhythmic activity related to motor imagery was investigated to explore the effects of motor imagery on brain function.

## 2. Materials and Methods

### 2.1. Subjects

Sixteen healthy right-handed subjects were chosen for this experiment. All subjects were between 20 and 25 years old and had no history of mental illness. The gender distribution of the recruited subjects is shown in Table 1. The experiment was approved by the Ethics Committee of Hebei University of Technology (HEBUThMEC2021025, 7 March 2021).

### 2.2. Experiment and Data Preprocessing

**Figure 1:**
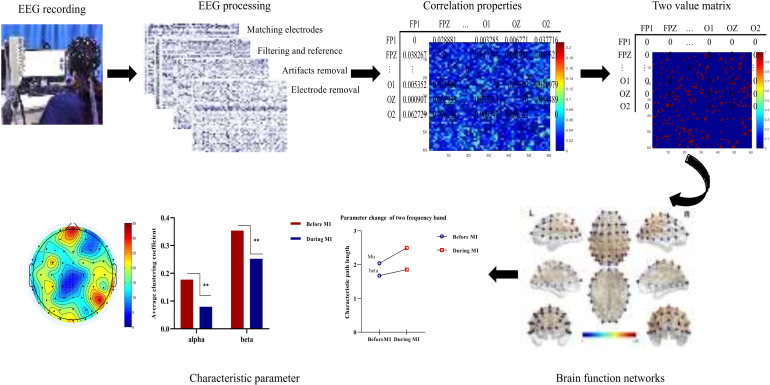
Overall research framework of this study.

**Figure 2:**
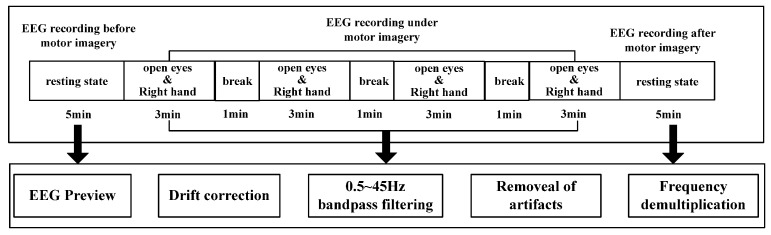
Experiment and data preprocessing.

In this study, subjects sat quietly 50 cm away from a screen, and the stimulus material (500 mL full drinks) was placed in the right-hand position, facing the table [14]. The subjects were asked to imagine repeatedly moving his/her right hand (i.e., grasping and lifting) in the state of open eyes. Data were collected for 20 minutes of movement imagination and five minutes of resting state for each subject. In each trail, the subjects first stared at the white “+” fixation point in the center of the screen and remained silent for the first two seconds, without any mental imagination. At two seconds, the computer emitted a short beep sound (for one second). Next, the imagination task began by indication marks. After three minutes, subjects rested for one minute, and this sequence was repeated four times. During the imagination task, subjects had to continuously imagine grasping and lifting with the right hand. The overall research framework is shown in Figure 1, and the experimental process and pretreatment are shown in Figure 2.

A Neuroscan EEG/ERP system (Neuroscan Ltd., Charlotte, NC, USA) was used to record EEG signals from 62 scalp positions, which were placed according to international 10–20 channel distribution. Ag/AgCl electrodes were used in this study. The electrodes were filled with conductive paste and attached to the skin of the head. Electrode impedance was kept below 10 kΩ. The reference electrode and ground electrode were placed according the Neuroscan4.3 user manual. Sample rate was 1000 Hz, and amplification factor was 500. The collected EEG signals need to be preprocessed to reduce artifacts caused by large amplitude (more than 50 μV), blinking and slow eye movement. After data preprocessing, EEG signals were analyzed using Matlab software (Mathworks Inc., Natick, MA, USA).

Data preprocessing mainly included matching electrodes, dedamaging electrodes, filtering (0.5~45.0 Hz), re-reference, artifact removal and frequency division. This process was completed by EEGLAB Toolkit of MATLAB software. As for the frequency-division operation, a filtering operation in EEGLAB was used, which adopts band-pass filtering. After frequency-division processing of EEG signals, mu (8–13 Hz) and beta (13–30 Hz) rhythms related to motor imagery were selected to construct and analyze the brain network. For data segmentation, random segmentation was used. Five groups of data segments were randomly selected for EEG data after artifact removal, and average processing was carried out to eliminate errors. This process took 10 s for each segment. 

### 2.3. Data Processing Based on Granger Causality

In recent years, methods based on nonlinear dynamics have been used to process and analyze EEG signals. Granger causality (GC) is a nonlinear analysis tool that can determine the direction of neuron interaction and can be used to measure effective connectivity [18]. It has been widely used to analyze EEG signals [19,20] and has been proven to play an important role in providing EEG change information [21,22].

The basic idea of GC analysis is to predict the effective connection between channels by relying on the variance obtained by the optimal least squares of all information data at a given past time point [23]. Over time, the cause overwhelms the effect. That is, for random time series *x*(*t*) and *y*(*t*), if *x*(*t*) contains the predicted information about the future value of *y*(*t*) and exceeds the past value that can be obtained from *y*(*t*) (and from other observed time series, *z*(1), *z*(2), etc.), then *x*(*t*) has a causal relationship with *y*(*t*) [24]. In the case of linear Granger causality (GC) and duality, *x*(*t*) and *y*(*t*) are assumed to be two randomly stationary time series, and the p-order univariate autoregressive (AR) model of *x*(*t*) and *y*(*t*) is evaluated as follows [25]:

(1) $$\begin{array}{c} x(t)=\sum_{\tau =1}^{p}a_{1\tau }x(t-\tau )+\sum_{\tau =1}^{p}b_{1\tau }y(t-\tau )+e_{1}(t)y(t)\\ =\sum_{\tau =1}^{p}a_{2\tau }x(t-\tau )+\sum_{\tau =1}^{p}b_{2\tau }y(t-\tau )+e_{2}(t) \end{array}$$

Formula (1) is the regression model, where $a_{1\tau }$, $b_{1\tau }$, $a_{2\tau }$ and $b_{2\tau }$ are the coefficients, and $e_{1}(t)$ and $e_{2}(t)$ are the prediction errors of each time series in the model.

(2) $$x(t)=\sum_{\tau =1}^{p}{\hat{a}}_{1\tau }x(t-\tau )+{\hat{e}}_{1}(t)$$

(3) $$y(t)=\sum_{\tau =1}^{p}{\hat{a}}_{2\tau }y(t-\tau )+{\hat{e}}_{2}(t)$$

Equations (2) and (3) are autoregressive models, where ${\hat{a}}_{1\tau }$ and ${\hat{a}}_{2\tau }$ are the estimation of single-variable AR coefficients of the p-order AR model, and ${\hat{e}}_{1}(t)$ and ${\hat{e}}_{2}(t)$ are the residual errors (prediction error) of the AR process. Compared with the prediction error of the autoregression model, if the variance of the prediction error in the regression model decreases significantly, then the time series *y*(*t*) has a causal relationship with *x*(*t*) [26]. Quantified Granger causality index (GCI) is:

(4) $$GCI_{y\to x}=\ln\frac{var({\hat{e}}_{1})}{var(e_{1})}$$

In Formula (4), $GCI_{y\to x}$ represents the proportion of signals from *y*(*t*) to *x*(*t*) in all signals flowing out of *x*(*t*), and the value range is [0,1]. The value is related to whether there is a causal relationship between the two EEG signal sequences. A value of 0 indicates that there is no connection between the two channels. The larger the value, the stronger the causal relationship. Similarly, Formula (4) is also applicable to the calculation of $GCI_{x\to y}$, this is $GCI_{x\to y}=\ln\frac{var({\hat{e}}_{2})}{var(e_{2})}$ [27]. 

On this basis, it is necessary to select the threshold value to establish the causal matrix and compare the relationship between GCI and the threshold value. If GCI is greater than the threshold value, there is a causal relationship between the two channels; otherwise, there is no causal relationship.

In this study, GC between the right-hand grasping motion imagination task group and the resting state was calculated and averaged, which was realized using MATLAB software. Then, the brain functional network was constructed according to the obtained average causality matrix.

### 2.4. Brain Functional Network Construction

Brain functional network is a measure of statistical connections between brain nodes or regions [28]. When the brain performs cognitive tasks related to imagination, changes are induced in the brain functional network, which can be reflected in the network structure [29]. This paper described the causal relationship between brain functional networks by drawing a directed graph to analyze the different states of brain functional networks during motor imagery. In this study, the region in which each electrode is located is defined as a network node for the collected 62-channel EEG signals, and the nodes needed to construct the network were obtained. The connection edge was determined by the value of GCI. The selection of appropriate thresholds played a decisive role in the construction of the brain functional network, and different thresholds directly affected the subsequent analysis of statistical characteristics of the network [30]. The principle of threshold selection proposed in this paper was to ensure the integrity of the network (that is, avoid too many isolated nodes). According to this principle, it was calculated that when the GC value between two nodes exceeds a threshold value (mu: 0.0650; beta: 0.0750), the node was connected.

### 2.5. Brain Functional Network Indicators

As a very effective theoretical analysis tool, complex networks can be used to describe various complex systems, including the constructed complex brain functional network based on EEG in different states of motor imagination tasks [31]. The statistical description of a complex network could further contribute to the study the influence of motor imagery tasks on brain functional networks. Therefore, the topological structure of brain functional networks and network characteristic statistics needed to be further discussed. In this study, brain functional networks under different states of motor imagination were constructed based on GCI values, and the network was quantitatively described using degree, clustering coefficient, characteristic path length and global efficiency [32]. The important statistical characteristic parameters selected are briefly described below.

- Degrees

The degree of a node is the number of edges connected between node *i* and node *j*. For a directed network, node degree is also divided into inputting degree and outputting degree, and the total degree of a node is the sum of inputting degree and outputting degree [16]. The greater the degree value, the higher the importance of nodes in the network. The node degree of node I is defined as [33]:

(5) $$k_{i}=\sum_{j\in N,i\neq j}a_{ij}$$

where $a_{ij}$ is the network matrix, and N is the set of all nodes in the network.

- Characteristic path lengh

The length of characteristic path is the average number of edges of the shortest path connecting two nodes, which is a key parameter to describe the internal information transmission of complex networks. Average path length is defined as [34]:

(6) $$L=\frac{1}{N(N-1)}\sum_{i,j\in N,i\neq j}d_{ij}$$

where N represents the number of nodes in the network, and $d_{ij}$ is the distance between nodes.

- Clustering coefficient

Clustering coefficient is an important parameter to measure the degree of intracerebral collectivization and connection tightness, which can be expressed as [35]:

(7) $$C_{i}=\frac{2E_{i}}{k_{i}(k_{i}-1)}$$

where $k_{i}$ is the number of nodes connected to node *i*, $E_{i}$ represents the actual number of edges between $k_{i}$ nodes connected to node *i*, and $k_{i}(k_{i}-1)/2$ represents the maximum number of possible edges between $k_{i}$ nodes connected to node *i*.

There are many nodes in a complex network, so the average clustering coefficient of the whole network is usually studied, i.e., the clustering coefficient of all nodes is averaged:

(8) $$C=\frac{1}{N}\sum_{i=1}^{N}C_{i}$$

where *C* is the average clustering coefficient of the entire network.

- Global efficiency

As a network efficiency, global efficiency represents the transmission capacity of the network. The larger the value, the lower the cost of energy or information transfer/exchange for network. This parameter is the average value of the reciprocal of the shortest path between each pair of nodes [36]:

(9) $$E_{global}=\frac{1}{N(N-1)}\sum_{i,j\in N,i\neq j}\frac{1}{l_{ij}}$$

where *N* represents the number of network nodes, and $l_{ij}$ is the length of the shortest path connecting two nodes, *i* and *j*.

## 3. Results

**Figure 3:**
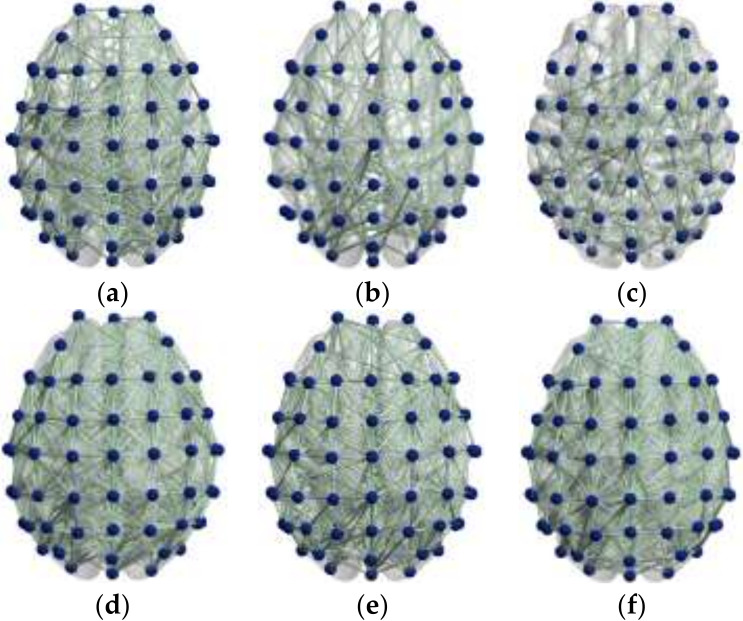
(**a**) Resting state before motor imagery task in the mu rhythm. (**b**) State during motor imagery task in the mu rhythm. (**c**) Resting state after motor imagery task in the mu rhythm. (**d**) Resting state before motor imagery task in the beta rhythm. (**e**) State during motor imagery task in the beta rhythm. (**f**) Resting state after motor imagery task in the beta rhythm.

Mu/beta wave is the main basic rhythm related to motor imagery. When people perform the motor imagination task, the EEG power and the corresponding brain activity of mu/beta rhythm will decrease [37]. In this study, mu/beta rhythm EEG data of 16 subjects were processed to explore the effects of motor imagery EEG on brain function. GCI values of each channel were calculated to analyze the difference in brain functional network connectivity during motor imagination. The results of brain functional network connectivity are shown in Figure 3.

It can be seen from Figure 3 that during the motor imagery task, the brain functional network of the mu rhythm brain region as a whole was significantly reduced, with prominent changes in the frontal lobe and primary motor area. Beta rhythm also showed decreased network connection, but the change was smaller than that in mu. Meanwhile, comparison of the two wavebands showed that the changes caused by motor imagery had a certain time delay, suggesting that motor imagery had an effect on the excitatory activation of the frontal lobe and sensorimotor cortex.

- Degrees

**Figure 4:**
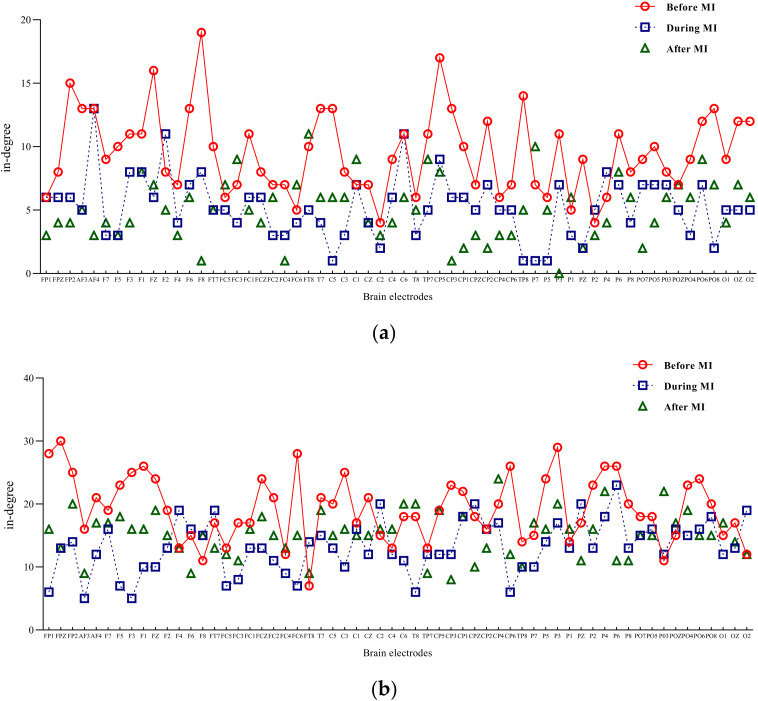
Mu/beta in degree of three different states of motor imagery. (**a**) Mu-rhythm in degree of three different states of motor imagery. (**b**) Beta-rhythm in degree of three different states of motor imagery.

**Figure 5:**
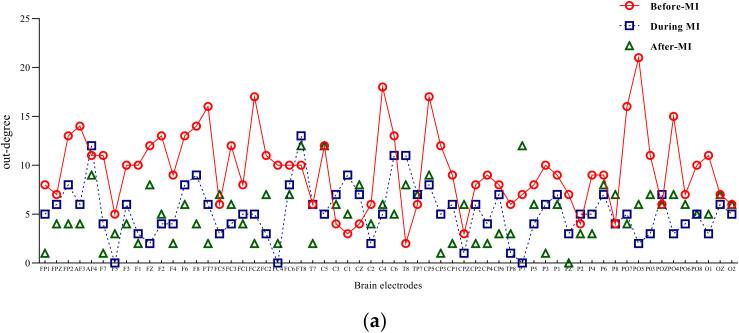

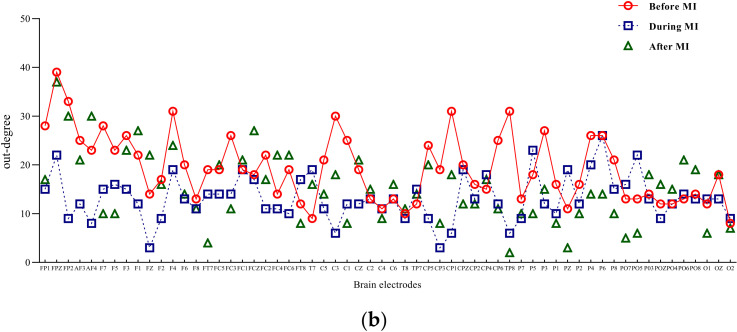
Mu/beta out degree of three different states of motor imagery. (**a**) Mu-rhythm out degree of three different states of motor imagery. (**b**) Beta-rhythm out degree of three different states of motor imagery.

Input degree refers to the flow of network information into a node, while out degree refers to the information flow out of a node. The degree of a node is the sum of input-degree and out degree of a node [18]. The two-band entry-degree and exit-degree line charts of three different states of motion imagination are shown in Figure 4 and Figure 5. Compared with the condition without motion imagination, the distribution trend of mu rhythm in motor imagery changed significantly, i.e., the nodes representing important causes and results changed significantly, and the distribution range was larger. Comparatively, the trend of beta-band access distribution was mainly concentrated in the frontal lobe region.

**Figure 6:**
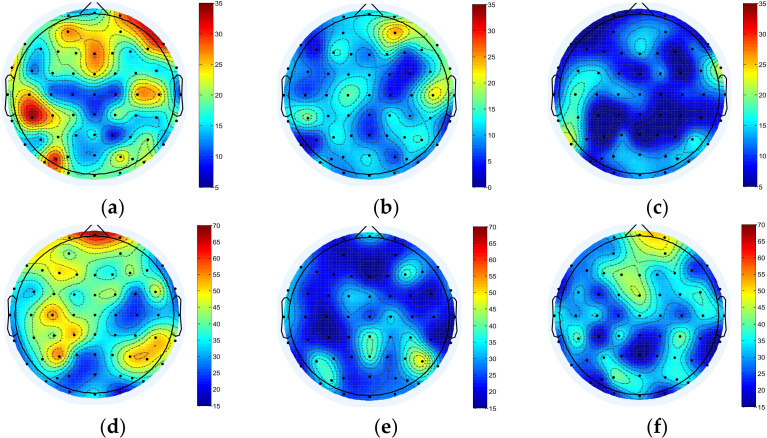
BIM of mu/beta in three different states of motor imagery. (**a**) BIM before motor imagery task in the mu rhythm. (**b**) BIM during motor imagery task in the mu rhythm. (**c**) BIM after motor imagery task in the mu rhythm. (**d**) BIM before motor imagery task in the beta rhythm. (**e**) BIM during motor imagery task in the beta rhythm. (**f**) BIM after motor imagery task in the beta rhythm. The color scale in the figure is generated based on the value of characteristic parameters of two different frequency bands.

Brain information mapping (BIM) is an EEG topographic map composed of nonlinear parameters of EEG signal [38], which can represent the changes of EEG signal characteristics. In this paper, the influence of motor imagination on brain function areas is explored by drawing the EEG information map using degree as parameter. The result is shown in Figure 6.

For mu/beta rhythm, the node degree of the whole brain decreased significantly during the motor imagery task, and the beta rhythm also decreased significantly. After the imagery task, mu rhythm further decreased, and beta rhythm increased to a certain extent.

A paired t-test was performed on the in degree and out degree of the two rhythms. Significant differences were found in mu/beta rhythm (*t* = −4.016; *p* < 0.05).

- Characteristic path lenght

Characteristic path length is an important parameter to measure brain functional networks. The higher its value, the lower the degree of network connection. In this paper, characteristic-path-length values of the mu/beta rhythm imagery task in three states were calculated, and the change rate of the characteristic path length in the moving imagery task compared with that in the resting state without the motion imagination task was calculated. The results are shown in Table 2.

**Figure 7:**
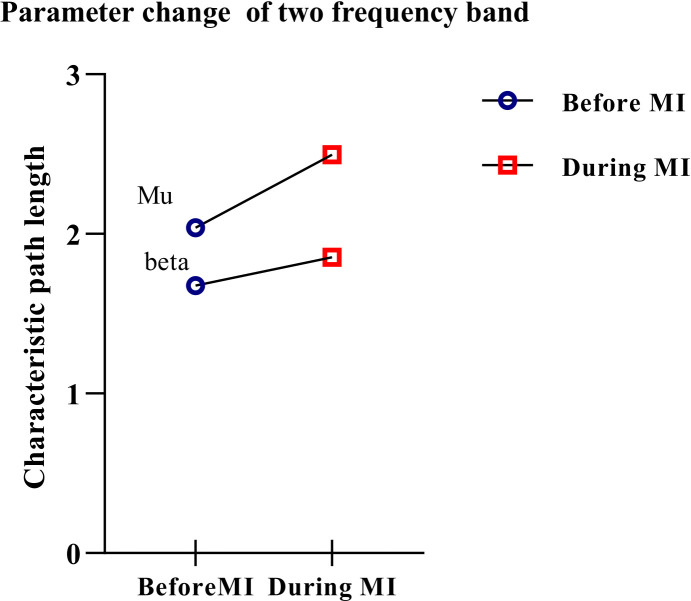
Variation of average characteristic path length.

The results showed that for mu/beta rhythm, when the participants performed the imagery task, the characteristics of the network path length increased to varying degrees. An increase in mu rhythms was apparent. Variations in average characteristic path length are shown in Figure 7.

A paired t-test is performed to calculate the difference in characteristic path length between the two rhythms. Significant differences were found in characteristic path length between before MI and during MI in mu and beta rhythms (*t* = 3.167; *p* < 0.05).

- Clustering coefficient

**Figure 8:**
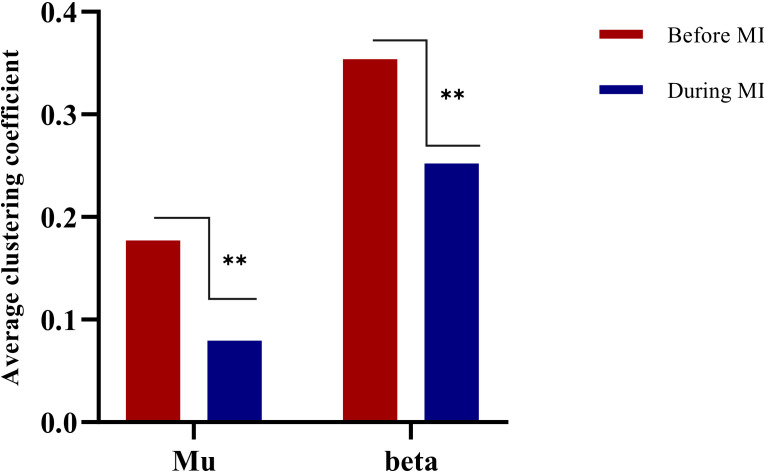
Histogram of average clustering coefficient change. ** means that there is a significant difference between two different states.

Clustering coefficient is related to the operating efficiency of a brain network. In this paper, the average clustering coefficients of mu/beta rhythm imagery tasks in two states were calculated, i.e., resting state before MI and the state during MI. A paired t-test was performed to calculate the difference in clustering coefficient between the two bands. Analysis focused on comparison of the resting state before performing the imagination task and during the imagination task, as shown in Figure 8.

The results showed that there was a significant difference between the two bands when all subjects performed the motor imagery task compared with the static state before the motor imagery task (*t* = −2.854; *p* < 0.05). There was a significant difference after the imagination task compared with before (t = −4.937; *p* < 0.05).

- Global efficiency

Global efficiency reflects the speed of information transfer in the brain functional network. The higher the global efficiency, the faster the speed of information transfer in the network. Average global efficiency of all subjects in the three experimental groups was calculated, and the specific results are shown in Table 3.

It can be seen from the results that when subjects engaged in motor imagery, the global efficiency of the brain functional network of mu rhythm was significantly reduced, and beta rhythm was reduced to a certain extent. A paired t-test was performed to calculate the difference in global efficiency between the two rhythms, The results showed that there were significant differences between the two bands when subjects performed the motor imagery task compared with the static state before the motor imagery task (*t* = −4.322; *p* < 0.05). There was no significant difference after the imagination task compared with before (*t* = −1.450; *p* > 0.05).

## 4. Discussion

From the perspective of the brain information-transmission mechanism, the brain functional network was constructed to explore the changes in brain activity during a motor imagery task. The results showed that mu/beta oscillations are consistently suppressed during motor imagery, although the specific effect remains to be confirmed. Data were collected five minutes after cessation of motor imagination and analyzed. The conclusion drawn is that the effect lasted for a certain period of time (18,000 ms). Specifically, the connections of the brain functional network with mu/beta rhythm become sparse. The degree distribution was also significantly reduced, and the information-transmission capacity and efficiency of the network were also inhibited. Changes were found in the whole brain region, especially in the frontal lobe and primary motor region. Functional brain activity is a nonlinear, dynamic behavior of neuron activity in some regions of the brain, and the mechanism of activation of cortical excitability through motor imagery is considered to be the decreased synchronicity of the basic neuron group [39]. This indicates that motor-imagery stimulation activates cortical excitability. 

The results obtained in this paper are consistent with the research results of Chen [40], who studied mu oscillation and motor-imagination performance and found that EEG activity under mu rhythm was reduced during the imagination task. Chen believed that the overall level of mu rhythm was a possible method to measure the success of individual motor-imagination performance. Daeglau [41] proposed the effect of activating event-related desynchronization induced by motor imagery through repeated practice of strengthening motor-imagery tasks. Matsumoto [42] investigated transcranial direct-current stimulation caused by motor imagery of synchronous adjustment and posited that the movement caused in imagining the physiological characteristics of synchronization is due to the change in the oscillation behavior of cortical neurons. Changes such as in the membrane potential and neurons of the primary motor area, according to the input signal, occur in response to the probability of response to imagination tasks. When receiving imaginary signals, the increase in cortical excitability and the depolarization of membrane potential of cortical neurons in relevant brain regions leads to more activation and desynchronization of neurons, which increases the ERD of mu rhythm. This view is more vividly illustrated by Jean [43] in research on the neurophysiological basis of motor imagery. In the process of motor imagery, the human cerebellum is activated, which reflects an inhibitory mechanism preventing efferent pulses triggered by imagination from reaching the medulla and muscle levels. Pickenhain [44] explained that the imagination of motor behavior may trigger part of the central nervous system. Therefore, the present study suggests that the triggering of inhibitory mechanisms forms an important part of the prefrontal cortex’s role during motor imagery.

In this study, similar results were obtained for beta rhythm and mu rhythm. This is consistent with the results of Jongsma [45], who found that the power of beta rhythm decreased most significantly in the sensory–motor region during motor imagination and that the energy of the alpha band seemed to decrease simultaneously with beta, which suggests that mu/beta rhythm has synergy. In addition, a Stolk [46] concluded similarly that the reduction in beta band energy is related to more specific motion activation. It can be seen from the above studies that motor imagination tasks can stimulate the mu/beta rhythm desynchronization phenomenon of EEG signal, especially in the sensorimotor area. From the perspective of functional brain network analysis of EEG signals, the influence of motor imagery on brain activity was explored in this paper. The results are consistent with other studies that discuss this influence from the perspective of energy, which indicate that, to some extent, motor imagery can stimulate a reduction in the synchronization of neurons in the brain, thus activating motor cortex excitability.

Study limitations: Only right-handed participants were included, and left-handed participants were not recruited. Shahid et al. [47] suggested that the estimation of hand dominance could affect the accuracy of motor imagery, which may be important for future research in this area.

Possible clinical applications and future perspectives: Motor impairment after stroke is a leading cause of disability. Motor imagery (MI), in addition to physical practice of rehabilitation tasks, in both right- and left-handed hemiplegic subjects, leads to enhanced functional recovery of paralyzed limbs among stroke sufferers. It is acknowledged that left- or right-hand MI is associated with a desynchronization (ERD) of mu (8–13 Hz) in contralateral EEG and with a synchronization (ERS) of beta rhythm (18–24 Hz) in ipsilateral EEG [48]. Therefore, this research provides a theoretical basis for the application of motor imagination as a means of clinical rehabilitation.

## 5. Conclusions

In this study, Granger causality was used to construct the brain functional network of a right-handed grasping motor-imagery task group, as well as the resting state. The degree, clustering coefficient, characteristic path length and global efficiency of mu/beta rhythm brain functional networks in the resting state were compared and analyzed. The results showed that mu/beta rhythm desynchronization could be induced by a motor imagery task, and the effect lasted for a certain period of time. The connectivity of mu/beta rhythm in the brain functional network became sparse, especially in the frontal lobe and primary motor area. At the same time, motor imagery was able to coordinate neuronal activity to activate cortical excitability. The combination of motor imagination and brain functional networks based on EEG can effectively reveal the mechanism of brain information transmission and provide an objective basis for the introduction of motor imagination as a restorative treatment in clinical practice. 

## Figures and Tables

**Table 1:** Basic information on subjects.

Variable	Subjects
Age/years	22.28 ± 5.81
Gender (male/female)	8/8

**Table 2:** Variation of average characteristic path length in three states of motor imagery in mu/beta rhythm.

Rhythm	Before MI	During MI	After MI
Mu	2.0398 ± 0.3251	2.4955 ± 1.0213	2.5805 ± 0.8697
Beta	1.6766 ± 0.6633	1.8534 ± 0.8635	1.7952 ± 0.5364

**Table 3:** Average global efficiency in three states of motion imagery in mu/beta rhythm.

Different States	Mu	Beta
Before MI	0.5474 ± 0.0125	0.6629 ± 0.2235
During MI	0.4181 ± 0.2101	0.5991 ± 0.3632
After MI	0.5060 ± 0.1298	0.6199 ± 0.2163

## Data Availability

The data presented in this study are available on request from the corresponding author. The data are not publicly available due to restrictions privacy.
